# Generalized models for estimating cerebral lateralisation of young children using functional transcranial Doppler ultrasound

**DOI:** 10.1002/hbm.70012

**Published:** 2024-09-04

**Authors:** Josephine E. Quin‐Conroy, Paul A. Thompson, Donna M. Bayliss, Nicholas A. Badcock

**Affiliations:** ^1^ School of Psychological Science University of Western Australia Western Australia Australia; ^2^ School of Education, Learning and Communication Sciences University of Warwick Coventry UK

**Keywords:** child, fTCD, generalized additive model (GAM), generalized linear model (GLM), laterality

## Abstract

Thompson et al., 2023 (Generalized models for quantifying laterality using functional transcranial Doppler ultrasound. *Human Brain Mapping*, 44(1), 35–48) introduced generalised model‐based analysis methods for determining cerebral lateralisation from functional transcranial Doppler ultrasound (fTCD) data which substantially decreased the uncertainty of individual lateralisation estimates across several large adult samples. We aimed to assess the suitability of these methods for increasing precision in lateralisation estimates for child fTCD data. We applied these methods to adult fTCD data to establish the validity of two child‐friendly language and visuospatial tasks. We also applied the methods to fTCD data from 4‐ to 7‐year‐old children. For both samples, the laterality estimates from the complex generalised additive model (GAM) approach correlated strongly with the traditional methods while also decreasing individual standard errors compared to the popular period‐of‐interest averaging method. We recommend future research using fTCD with young children consider using GAMs to reduce the noise in their LI estimates.

## Graphical abstract

We tested recent statistical methods for quantifying the lateralisation of cognitive functions as measured by fTCD. We found that using a generalized additive model approach decreased individual standard error estimates in the laterality estimates for adult data collected using child‐friendly tasks and for data collected from young children (4–7 years).

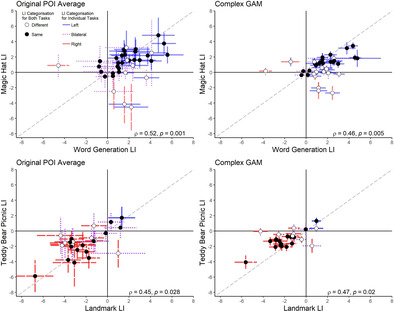

## 1 INTRODUCTION

Cerebral lateralisation refers to the observation that many cognitive functions, such as language and visuospatial processing, tend to occur more in one hemisphere of the brain than the other (Hervé et al., 2013). Research on lateralisation relies on a number called a laterality index (LI) to quantify this lateralisation of cognitive functions. Most often, a positive number indicates leftward lateralisation and a negative number indicates rightward lateralisation. Additionally, large LIs indicate stronger lateralisation and smaller LIs indicate weaker lateralisation, with LIs close to zero denoting little or no bias towards either hemisphere. LIs are generated by measuring hemispheric differences whilst participants perform a task that evokes a particular cognitive function. For example, during the period of interest (i.e., the period of a task where task‐related activation is expected to occur; POI) of a language task, brain activity is typically lateralised to the left hemisphere of the brain (Carey & Johnstone, 2014; Knecht et al., 1998). Conversely, during a visuospatial task, brain activity is typically lateralised to the right hemisphere of the brain (Vingerhoets & Stroobant, 1999; Whitehouse et al., 2009).

Cerebral lateralisation is often determined using functional magnetic resonance imaging (fMRI). FMRI is an imaging technique that estimates blood flow and blood oxygenation as a proxy for brain activity; localised neuronal activity causes an increase in local blood flow to the activated region (a phenomenon known as neurovascular coupling), which causes a change in relative blood‐oxygen level dependent (BOLD) signals measured by fMRI (Hillman, 2014). Researchers typically count activated voxels within homotopic regions in each hemisphere to determine which hemisphere is more active during a given cognitive task, therefore determining the hemisphere the cognitive function is lateralised to. FMRI has good spatial resolution, but is expensive to administer and requires participants to lie still for the duration of the cognitive tasks. In addition, methodological issues such as the use of statistical thresholds to determine active voxels in each hemisphere can make measuring weaker cerebral lateralisation problematic using fMRI (see Somers et al., 2011).

One alternative to fMRI is functional transcranial Doppler ultrasound (fTCD). FTCD is a noninvasive neurophysiological technique which uses two Doppler ultrasound probes to measure cerebral blood flow velocity by insonating the left and right middle cerebral arteries (MCAs) through the temporal bone windows (Lohmann et al., 2006). Just as with fMRI, fTCD estimates brain activity based on neurovascular coupling resulting from neuronal activity; however, fTCD only measures blood flow velocity in the MCAs, meaning fTCD can be used to estimate the brain activity in regions supplied by the MCAs in each hemisphere. The MCAs supply blood to approximately 50% of each hemisphere, including regions of the frontal, temporal, and parietal lobe associated with language functions and visuospatial processing (Gibo et al., 1981; van der Zwan et al., 1993). Cerebral lateralisation as determined by fTCD is strongly correlated with the gold‐standard Wada test (Knecht et al., 1998) and fMRI (Deppe et al., 2000; Jansen et al., 2004; Somers et al., 2011). Compared to fMRI, fTCD is relatively inexpensive, unobtrusive, and robust to small head movements during recording, making it an appealing choice amongst researchers investigating cerebral lateralisation, particularly for child participants.

As fTCD only records data from two channels, one for each hemisphere, researchers typically determine LIs by averaging the difference between the left and right channels during the POI. This differs from the analysis of fMRI data quite substantially, given that fMRI measures the blood‐oxygen‐level‐dependent signals from each voxel throughout the brain in a scale of millimetres, therefore requiring more sophisticated statistical techniques to sift through the activation data to determine lateralisation. A recent paper by Thompson et al. (2023) proposed a new method of processing fTCD data that more closely aligns with the statistical analysis of fMRI data. The authors tested three new methods using either a generalised linear model (GLM) approach, a “simple” generalised additive model (GAM) approach, or a “complex” GAM approach to model the fTCD blood flow velocity. Thompson et al. reported that, while all three methods produced similar LIs to the POI averaging method, the complex GAM method substantially decreased the error for LIs at the individual level, allowing for a more precise estimate of lateralisation.

Research in lateralisation has shown interest in determining if the development of language and visuospatial lateralisation is related to cognitive ability in children, particularly if atypical lateralisation is a predictor of worse language ability (Bartha‐Doering et al., 2018; Bishop et al., 2014; Kohler et al., 2015). As the lateralisation of language occurs below the age of 5 years (Weiss‐Croft & Baldeweg, 2015), being able to reliably and accurate determine lateralisation in young children would be beneficial to the field, and fTCD as a technique is well suited for this purpose. However, working with young children in any neurophysiological context can present challenges. Young children are prone to fidgeting during recordings and speaking or disengaging during trials, which results in fewer acceptable epochs included in the analysis (e.g., Badcock et al., 2018). In addition, our recent child‐friendly tasks for measuring language and visuospatial lateralisation showed greater uncertainty for the LIs with an adult sample compared to the gold‐standard lateralisation tasks, the word generation and landmark tasks; this was attributed to the increased visual and audio stimulation of the child‐friendly tasks, which is necessary to maintain the attention of a three‐year‐old (Quin‐Conroy et al., 2022). Due to this increased chance of measurement error, the prospect of using a data processing method that reduces standard error is attractive.

The aim of this paper is to establish the suitability of the GLM and GAM methods for increasing the reliability of fTCD lateralisation data collected using child‐friendly tasks. To do this, we will first evaluate these methods for analysing fTCD data for child‐friendly language and visuospatial tasks with adults (previously reported by Quin‐Conroy et al., 2022). Then, we will assess their use with fTCD data collected from a sample of young children aged 4‐ to 7‐years old to verify that the new methods do indeed increase the precision (i.e., decrease the individual standard errors) of their laterality estimates compared to the traditional POI averaging method.

## 2 MATERIALS AND METHODS

### 2.1 Participants

#### 2.1.1 Adult dataset

The data used for the adult sample of the current paper has been previously reported in Quin‐Conroy et al. (2022). The sample consisted of 37 adults between the age of 17 and 51 years (M = 23 years; female = 25, male = 12) who spoke English as a first language and had no known neurological conditions (e.g., epilepsy). Thirty participants were self‐reported right‐handers, and seven were self‐reported left‐handers. Of the full sample, 36 participants completed both language tasks (i.e., the adult and child‐friendly tasks) with 10 or more acceptable fTCD epochs, and 24 participants completed both visuospatial tasks with 10 or more acceptable epochs (see Quin‐Conroy et al., 2022, for more information on the sample). The data from this sample was collected with approval by the University of Western Australia Human Research Ethics Committee (RA/4/20/6096).

#### 2.1.2 Child dataset

The child sample consisted of 56 participants between the ages of 4 to 7 years (M = 5.4 years) who spoke English as a first language and had no known neurological conditions. Participants were recruited using an online advertisement. Eight additional participants were recruited but their data is not reported here due to technical error (*n* = 2) or because the participant did not want to wear the fTCD headset (*n* = 6). Of the remaining sample, 50 completed the child‐friendly language task (i.e., the magic hat task) with 8 or more acceptable fTCD epochs, and 50 completed the child‐friendly visuospatial task (i.e., the teddy bear picnic task) with 8 or more acceptable fTCD epochs. Forty‐nine participants were right‐handed and seven were left‐handed according to an adapted version of the Edinburgh Handedness Inventory (Bishop et al., 2014). The data from this sample was collected with approval by the University of Western Australia Human Research Ethics Committee (2020/ET000061).

### 2.2 Materials

For both samples, we used a DWL Multidop T device or a DWL Doppler‐Box device (DWL Elektronische Systeme, Singen, Germany) and a Diamon headset with two 2 MHz probes to measure blood flow velocity in the left and right MCAs simultaneously whilst participants completed several computer tasks. As reported in Quin‐Conroy et al. (2022), participants in the adult dataset completed two language tasks: the word generation task, and a child‐friendly magic hat task. Participants also completed two visuospatial tasks: the landmark task, and the child‐friendly teddy bear picnic task. Participants completed a maximum of 20 trials of the word generation, landmark, and teddy bear picnic tasks and 25 trials for the magic hat task.

For each trial of the word generation task, participants were shown a blank screen for 35 s, then the words “Clear mind” for 5 s. Participants were then shown one of 20 letters of the alphabet for 15 s, during which time they were instructed to silently generate words beginning with that letter. Then, participants were given 20 s to say aloud the words that they had just silently generated.

For each trial of the magic hat task, participants were shown a 19 s animation of a cartoon face with a top hat moving up and down the screen before its hat fell to the bottom half of the screen and a voice said “Look.” Then a 12 s picture naming period began, wherein the hat would be replaced by four images of innocuous objects in succession for 3 s each. Each image was accompanied with a voice asking what the object was (e.g., “What's this?”). A total of 100 objects were shown once in randomised order. Following the picture naming period was another animation lasting 9 s of a celebratory sound and the cartoon face making a “shh” motion accompanied by the voice saying “Shh.”

Both visuospatial tasks involve making line bisection judgements. For each trial of the landmark task, the participants were first shown a blank screen for 30 s, then the words “Clear mind” for 5 s. The line bisection period of the trial involved six judgements of whether a landmark (i.e., a small vertical line) bisected a longer horizontal line at its midpoint or not. Each landmark stimulus was shown for 200 ms, followed by masks consisting of randomly generated black and white lines. Participants were given 1700 ms for each landmark judgement to indicate via button press if the landmark bisected at the true midpoint or not.

For each trial of the teddy bear picnic task, participants were shown a black screen for 10 s accompanied by a voice saying “Shh.” A plate would then appear in the middle of the screen surrounded by six food items of a particular type (e.g., cupcakes) as the voice labelled the food type (e.g., “Let's cut up some cupcakes”). Then, for the line bisection period of the trial, participants were shown one of the food images on the plate. A knife flashed over the food image for 100 at 500 ms intervals. For each flash, the knife moved horizontally to a random non‐middle location alternating to the left and right of the midpoint before moving to the midpoint, at which point the knife would continue flashing in place until the participant pressed the button. For each food image, the knife would either begin at a non‐middle point and move 1–5 times before being shown at the midpoint, or it would start at the midpoint and not move horizontally. Once a correct button press was made (i.e., a button press when the knife was at the midpoint), a chopping sound played for 400 ms, and the next food image would show. Participants were not instructed to press the button with a specific hand to avoid overloading young children with instructions; this is not believed to affect lateralisation estimates significantly. Once 12 s passed from the beginning of the line bisection period, no new food images were presented. Instead, the participant was shown a 12 s animation of a teddy bear moving onto the screen, eating one of the foods from the previous line bisection period, and celebrating, ending with a “Shh” sound as the screen fades to black again. The task includes 18 different food types in total shown in randomised order.

For the child dataset, the fTCD procedure and tasks were identical to that for the adult dataset, except that the child participants only completed the magic hat task and teddy bear picnic task (in that order). Some minor changes in timing were made to the versions of the child‐friendly tasks used for the child participants—the images in the magic hat task were shown for 4 s each (instead of 3 s), the knife in the teddy bear picnic was shown at each location for 1 s at 1.5 s intervals (instead of 100 ms for 500 ms intervals), and the black screen in the teddy bear picnic task was only shown for 7 s and accompanied by snoring sounds. In addition, children only completed a maximum of 18 trials for each task to reduce the duration of the tasks. Individual trials were removed if the child was disengaged with the task or spoke during the baseline period (or if they spoke during the food cutting period during the teddy bear picnic task). The baseline period for both tasks was −8 to −3 s relative to the event marker at the beginning of the stimulus; the POI for both tasks was 5–20 s relative to the marker. This is longer than the adult POIs for the tasks, which were 5–15 s, as previous research has shown that young children have slower responses to stimuli in similar fTCD tasks (Badcock et al., 2018).

### 2.3 fTCD data analysis

To recreate Thompson et al.'s (2023) analyses, we estimated the LIs for the fTCD datasets using four methods from their paper—the POI averaging method, the GLM method, the simple GAM method, and the complex GAM method. For the adult fTCD dataset, we also included the LIs estimated using the POI averaging method of Quin‐Conroy et al. (2022), which includes slightly different preprocessing steps, for comparison (referred to as the “original POI method” henceforth and described at the end of this section). The fTCD data for both datasets were processed in R using scripts based on those made available by Thompson et al. (2023) through Open Science Framework (OSF; https://osf.io/gw4en/), with alterations to the preprocessing steps, described below. See Thompson et al. (2023) for a more in‐depth description of the statistical underpinnings of these methods.

In line with the preprocessing steps for all of Thompson et al.'s (2023) methods, the raw data was down sampled from 100 to 25 Hz. Extreme high or low points in the data (determined as data above the 99.99 or below the .01 quantiles for the participant) were removed and replaced using cubic spline interpolation. Heartbeat integration was performed by detecting peaks in the data that were spaced as genuine heartbeats (i.e., equal to or greater than 0.48 s apart, corresponding with a maximum expected heartrate of 125 beats per minute) and then averaging the data between peaks. Epochs were then segmented relative to the event markers for each trial and normalised so that the mean of the left and right channels was 100. Outliers (i.e., data points above or below 3 standard deviations from the mean) were removed. Epochs were baseline corrected according to a baseline period of −10 to −5 s for the adult tasks and a baseline period of −8 to −3 s for the child‐friendly tasks for both datasets.

The POI averaging method described by Thompson et al. (2023) estimates the LI as the average difference between the left and right activation within the POI window.

For the GLM method, a hemodynamic response function (HDR) was estimated using the *fmri* R package (Tabelow & Polzehl, 2011). The model uses the following equation:

(1) $$y=\begin{array}{l} \beta_{0}+\beta_{1}\cdot \mathrm{HDR}+\beta_{2}\cdot \text{hemisphere}+\beta_{3}\cdot \mathrm{HDR}*\text{hemisphere}\\ +\;\beta_{4}\cdot t+\beta_{5}\cdot t^{2}+\beta_{6}\cdot t^{3}+\varepsilon \end{array}$$

where *y* is observed fTCD activation, and *t* is time in seconds from the beginning of the task. The predictors were the HDR (*β*_1_), hemisphere (*β*_2_; coded as 1 for activation measured from the left probe and −1 from the right probe), the interaction between hemisphere and HDR (*β*_3_), and time from the start of the experiment as linear (*β*_4_), quadratic (*β*_5_), and cubic terms (*β*_6_) to account for change in the signal throughout the recording session (Worsley et al., 2002). These predictors are included in the model as follows: the observed fTCD activation. The LI is the coefficient for the interaction term between hemisphere and HDR ($\beta_{3}$).

The simple and complex GAM methods uses GAM from the *mgcv* R package (Wood, 2017). The GAM approach is similar to GLM but can fit nonlinear trends using smooth functions which can include parametric and nonparametric terms (Pedersen et al., 2019). As Thompson et al. (2023) describe, the GAM method allows for the HDR to be directly estimated from the data and for the inclusion of more predictors in the model, which should increase the precision of the LIs. The GAM methods, unlike the GLM method, require specification of the POI window within the epoch. Both GAM methods used the fTCD activation as the predictors. The simple GAM model is

(2) $$y=\begin{array}{l} \beta_{0}+\beta_{1}\cdot s\left(t\right)+\beta_{2}\cdot s\left(r\right)+\beta_{3}\cdot \mathrm{POI}+\beta_{4}\cdot \text{hemisphere}+\beta_{5}\cdot \mathrm{POI}\\ {}*\;\text{hemisphere}+\varepsilon , \end{array}$$

where *y* is observed fTCD activation, *s* is a smoother, *t* is time from the beginning of the task, and *r* is the relative time within the epoch. The simple GAM model includes POI (*β*_3_; coded as a boxcar function where 1 is the POI and 0 is not), hemisphere (*β*_4_), the interaction between POI and hemisphere (*β*_5_), the time from the start of the experiment (*β*_1_), and the relative time within the epoch (*β*_2_) as predictors. The complex GAM method is shown in the following equation:

(3) $$\begin{array}{c} y=\beta_{0}+\beta_{1}\cdot s\left(r\right)+\beta_{2}\cdot s\left(r,\text{epoch}\right)+\beta_{3}\cdot \mathrm{POI}+\beta_{4}\cdot \text{hemisphere}\\ +\;\beta_{5}\cdot \mathrm{POI}*\text{hemisphere}+\varepsilon , \end{array}$$

which uses the same predictors but adds epoch as a factor of the relative time (*β*_2_), meaning that smooth functions are replicated for each epoch separately. This allows for the variation across epochs to be included in the model, reducing the within‐individual error. Both GAM methods determine the LI as the coefficient of the interaction between hemisphere and POI (*β*_5_).

The adult fTCD data as processed using the original POI method detailed in Quin‐Conroy et al. (2022) was also included as a comparison to Thompson et al.'s (2023) methods. The original POI method calculates the LI as the difference between left and right signals during the POI (identical to Thompson et al.'s POI averaging method); however, the preprocessing steps were done using the original MATLAB scripts (available on OSF at https://osf.io/62m3u/). Briefly, these steps involved: linear correction for heart cycle patterns; correcting extreme values beyond −3 and 4 standard deviations using MATLAB's “linspace” function; extraction of epochs relative to event markers; normalisation of the data to a mean of 100; rejection of extreme values; baseline correction; and finally the calculation of the LI as the mean left‐minus‐right blood flow velocity difference within the POI.

For all methods, positive LIs indicate leftward lateralisation and negative LIs indicate rightwards lateralisation. Participants were categorised as bilateral for a task if the 95% confidence interval for their LI overlapped with zero. All data and scripts used for this paper are available on OSF at https://osf.io/2n5gj/.

### 2.4 Evaluation of GLM/GAM methods

To evaluate the GLM and GAM methods for the child‐friendly tasks, first we ensured that the methods are appropriate for both language and visuospatial tasks in our adult dataset. As in Thompson et al. (2023), for each task we assessed lateralisation at the group level (as language tasks are expected to be left‐lateralised and visuospatial are expected to be right‐lateralised), the within‐subjects SE of the LI, the *R* ^2^ of the model, the Akaike information criterion (AIC), and the Bayesian information criterion (BIC). Lower within‐subjects SEs result in smaller confidence intervals, thus decreasing the number of people labelled as bilateral due to noise rather than true bilaterality. The *R* ^2^ is a measure of the proportion of variance accounted for by each model, with a higher number indicating more variance explained by the predictors. The AIC and BIC estimate the prediction error in each model and can be used to compare models of differing complexity to assess relative goodness of fit, with smaller values indicating better fit.

We then used the adult dataset to test the validity of the child‐friendly tasks against the gold standard adult tasks when using the method with the best fit, which is expected to be the complex GAM method based on Thompson et al.'s (2023) findings. We did this by correlating the adult and child‐friendly task LIs using the Spearman method to reduce the impact of extreme LIs (Badcock et al., 2018). To replicate the validity check in Quin‐Conroy et al. (2022), we required a Spearman's rho of at least .40 (i.e., medium strength) to establish validity between the tasks.

Finally, we applied Thompson et al.'s measures of fit detailed above to the child dataset to evaluate the appropriateness of the processing methods (in particular, the complex GAM method) for use with fTCD data from young children.

## 3 RESULTS

### 3.1 Adult dataset

#### 3.1.1 Language tasks

**FIGURE 1:**
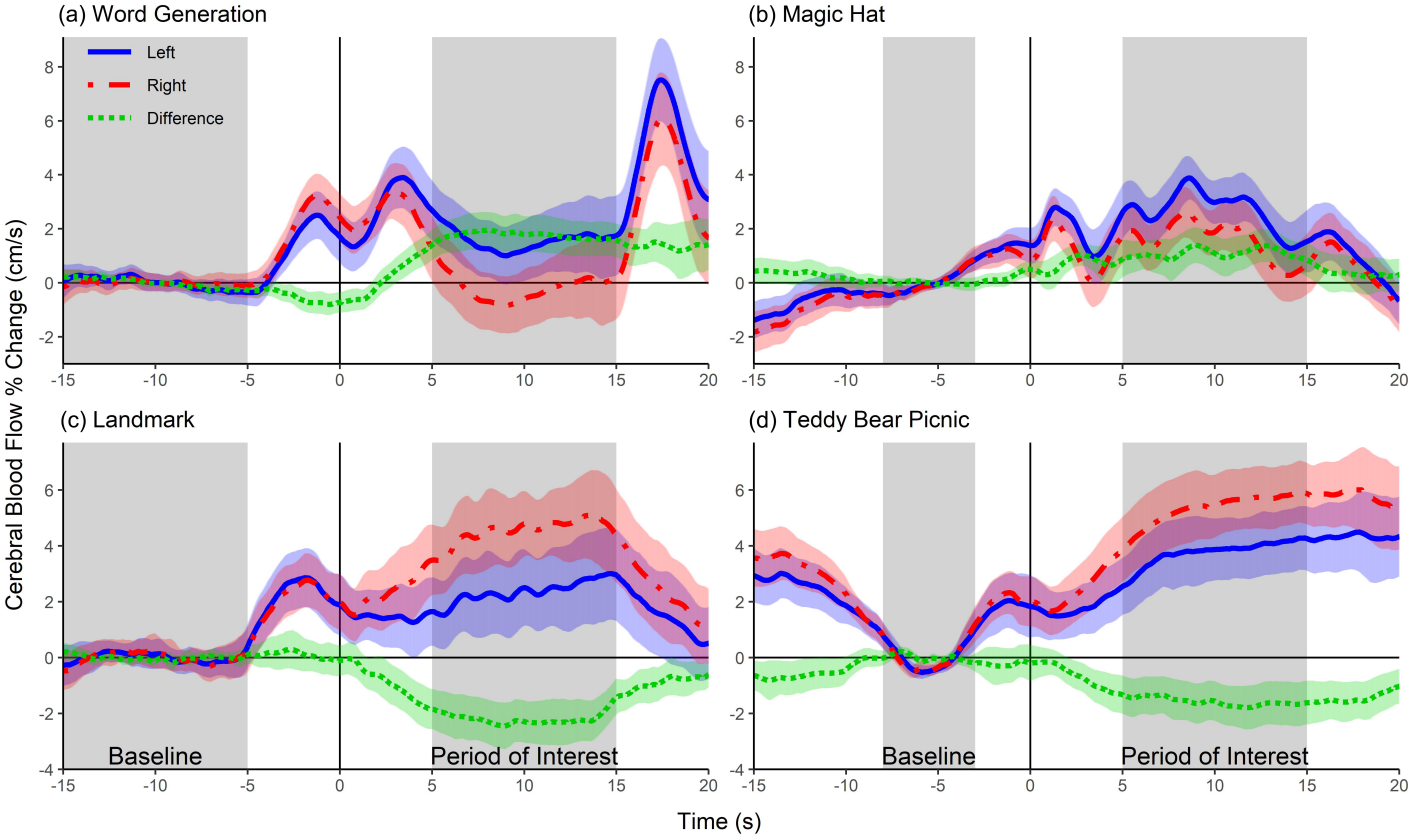
Group‐averaged change in blood flow velocity for the adult dataset. Blood flow velocity change for the left (unbroken blue line) and right (dashed red line) middle cerebral arteries for the word generation task (a), the magic hat task (b), the landmark task (c) and the teddy bear picnic task (d). The left‐minus‐right difference is shown as the green dotted line. Shaded ranges represent the 95% CI. The period of interest (POI) and baseline period (used for the POI averaging method) are shown in grey. Reprinted with permission from Quin‐Conroy et al. (2022), http://www.tandfonline.com.

**FIGURE 2:**
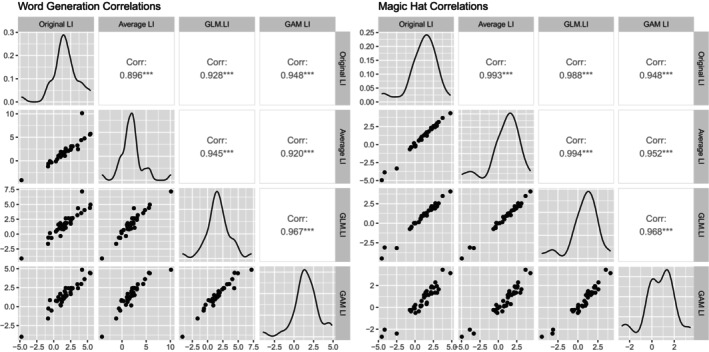
Correlations matrices, scatterplots and density plots comparing different processing methods for the language tasks for adult dataset. GAM, generalised additive model; GLM, generalised linear model; LI, laterality index. Original LI refers to LIs from the original period of interest (POI) averaging method detailed in Quin‐Conroy et al. (2022), and Averaging LI refers to POI averaging method as detailed by Thompson et al. (2023). “GAM LI” refers to both the simple and complex GAM LIs, as the methods generate identical LIs but different standard errors of the LIs.

Group‐averaged blood flow velocity waveforms for all four adult tasks, as published by Quin‐Conroy et al. (2022), is shown in Figure 1. Figure 2 displays the correlation matrices of the LIs for the word generation and magic hat tasks. The LIs generated by the original POI averaging method correlate strongly with the new methods for both tasks (all *r* > .9). Table 1 displays the indicators of model fit chosen by Thompson et al. (2023) for the word generation and magic hat tasks, respectively. Note that the simple and complex GAM methods generate identical LIs but different standard error of the LIs. The GLM and GAM methods gave smaller mean LIs; however, both language tasks showed significant left lateralisation at the group level for all methods (i.e., no confidence intervals overlapped with zero). In line with the findings of Thompson et al. (2023), the complex GAM method produced the smallest standard error of individual LIs, with the complex GAM mean standard errors being half that of the original method's standard error for both tasks. The complex GAM method also had the best model fit according to the *R* ^2^, AIC, and BIC values out of the GLM/GAM models for both language tasks. The complex GAM method categorises participants similarly to the original averaging method, albeit with fewer bilateral participants. This is expected, given that the smaller standard errors would mean that participants are less likely to have 95% confidence intervals overlap with zero.

**TABLE 1:** Mean (95% CI) for indicators of model fit for the language tasks for adult dataset.

Indicators of model fit	Original method	POI average method	GLM method	Simple GAM method	Complex GAM method
Word generation					
Mean LI	1.69 [1.07, 2.31]	1.97 [1.22, 2.72]	1.71 [1.07, 2.36]	1.53 [0.99, 2.06]	1.53 [0.99, 2.06]
Within‐subject SE LI	0.67 [0.61, 0.74]	0.95 [0.64, 1.26]	0.67 [0.58, 0.75]	0.63 [0.55, 0.71]	0.39 [0.32, 0.46]
*R* ^2^	NA	NA	0.05 [0.04, 0.06]	0.17 [0.14, 0.20]	0.67 [0.63, 0.72]
AIC	NA	NA	11,427 [10,611, 12,243]	11,171 [10,369, 11,974]	9534 [8765, 10,304]
BIC	NA	NA	11,476 [10,660, 12,292]	11,254 [10,450, 12,058]	10,387 [9618, 11,157]
% Right	2.7	2.7	2.7	2.7	5.4
% Bilateral	35.1	40.5	35.1	35.1	18.9
% Left	62.2	56.8	62.2	62.2	75.7
*r* with POI average	NA	0.90 [0.8, 0.95]	0.93 [0.86, 0.96]	0.95 [0.9, 0.97]	0.95 [0.9, 0.97]
Magic hat					
Mean LI	1.08 [0.45, 1.71]	1.01 [0.38, 1.64]	0.84 [0.28, 1.40]	0.69 [0.26, 1.12]	0.69 [0.26, 1.12]
Within‐subject SE LI	0.77 [0.65, 0.89]	0.75 [0.64, 0.85]	0.60 [0.55, 0.65]	0.56 [0.51, 0.61]	0.30 [0.27, 0.33]
*R* ^2^	NA	NA	0.05 [0.04, 0.07]	0.11 [0.09, 0.13]	0.73 [0.69, 0.76]
AIC	NA	NA	11,070 [10,349, 11,791]	10,966 [10,247, 11,686]	8908 [8295, 9521]
BIC	NA	NA	11,119 [10,397, 11,841]	11,043 [10,322, 11,765]	9970 [9321, 10,618]
% Right	8.1	8.1	8.1	8.1	10.8
% Bilateral	45.9	45.9	48.6	48.6	37.8
% Left	45.9	45.9	43.2	43.2	51.4
*r* with POI average	NA	0.99 [0.99, 1]	0.99 [0.98, 0.99]	0.95 [0.9, 0.97]	0.95 [0.9, 0.97]

Abbreviations: AIC, Akaike information criterion; BIC, Bayesian information criterion; GAM, generalised additive models; GLM, generalised linear models; LI, laterality index; POI, period of interest; SE, standard error.

**FIGURE 3:**
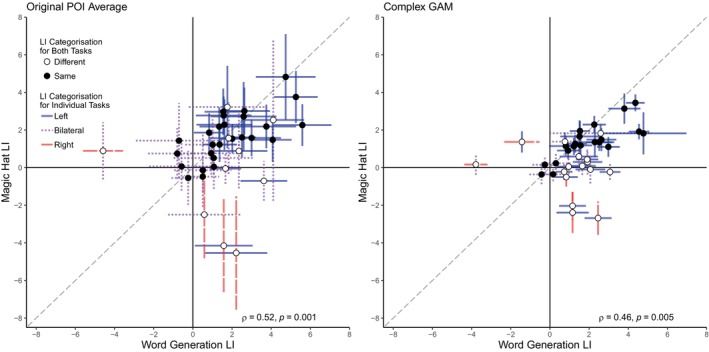
Scatterplots of laterality indices from the POI averaging and complex GAM methods for the language tasks from the adult dataset. GAM, generalised additive models; POI, period of interest. Scatterplots show the relationship between the word generation and magic hat LIs for the adult dataset when estimated using the original POI averaging method (left) and the complex GAM method (right). Spearman correlations and *p*‐values for both plots are included. Error bars show the 95% CI for each participant, coded by colour and pattern to indicate categorisation of each LI as either left‐lateralised (unbroken blue line), right‐lateralised (dashed red line), or bilateral (dotted purple line). Black circles indicate that the adult and child‐friendly task have the same categorisation, and white circles indicate different lateralisation categories for the two tasks.

Figure 3 presents scatterplots of the relationship between the word generation and magic hat LIs for the original POI averaging method and the complex GAM method. The Spearman correlation between LIs for the two tasks using the original method was significant, *ρ*(34) = .52, *p* = .001; the correlation using the complex GAM method is also significant, *ρ*(34) = .46, *p* = .004. After categorising participants for both tasks, 72.2% of participants (*n* = 26) have the same categorisation for both tasks using the original POI averaging method, whereas only 55.6% of participants (*n* = 20) have the same categorisation for both tasks using the complex GAM method. The Spearman correlation between word generation and magic hat when categorised according to the complex GAM method (coded as 1 for left lateralised, 0 for bilateral, and −1 for right lateralised) was not significant, *ρ*(34) = .08, *p* = .646. This may be due to the eight participants categorised as left lateralised in the word generation task but bilateral for the magic hat task.

#### 3.1.2 Visuospatial tasks

**FIGURE 4:**
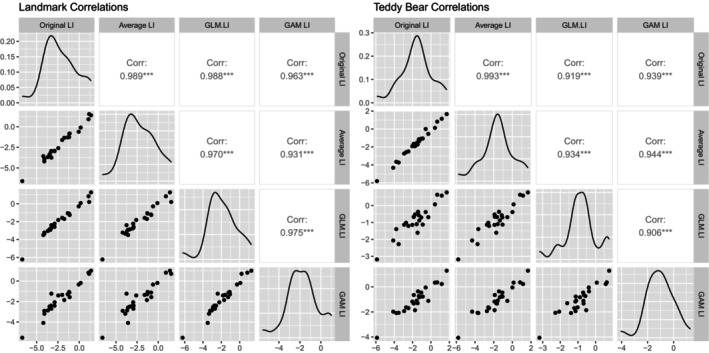
Correlations matrices, scatterplots and density plots comparing different processing methods for the visuospatial tasks for adult dataset. GAM, generalised additive model; GLM, generalised linear model; LI, laterality index. Original LI refers to LIs from the original period of interest (POI) averaging method detailed in Quin‐Conroy et al. (2022), and Averaging LI refers to POI averaging method as detailed by Thompson et al. (2023).

Figure 4 presents the correlation matrices of the LIs for the landmark and teddy bear picnic tasks, and Table 2 displays the indicators of model fit chosen for the landmark and teddy bear picnic tasks, respectively. The LIs generated by the original POI averaging method correlate strongly with the new methods for both tasks (all *r* > .9). Mean LIs show significant rightwards lateralisation at the group level for all methods for both visuospatial tasks. Again, the complex GAM method produced the smallest standard error of individual LIs—at less than half the mean standard errors compared to the original method—and had the best model fit according to the *R* ^2^, AIC, and BIC values out of the GLM/GAM models for both tasks. As with the language tasks, the complex GAM method decreased the number of participants categorised as bilateral for visuospatial processing, although the distribution of left, right, and bilateral participants is similar between the methods.

**TABLE 2:** Mean (95% CI) for indicators of model fit for the visuospatial tasks for adult dataset.

Index	Original method	POI average method	GLM method	Simple GAM method	Complex GAM method
Landmark					
Mean LI	−2.19 [−2.83, −1.56]	−2.13 [−2.76, −1.50]	−1.89 [−2.42, −1.37]	−1.76 [−2.25, −1.26]	−1.76 [−2.25, −1.26]
Within‐subject SE LI	0.70 [0.62, 0.77]	0.73 [0.66, 0.80]	0.58 [0.53, 0.62]	0.55 [0.51, 0.59]	0.31 [0.29, 0.34]
*R* ^2^	NA	NA	0.09 [0.06, 0.11]	0.18 [0.14, 0.22]	0.72 [0.69, 0.76]
AIC	NA	NA	10,800 [10,232, 11,367]	10,608 [10,042, 11,174]	8771 [8274, 9268]
BIC	NA	NA	10,849 [10,281, 11,416]	10,689 [10,122, 11,256]	9669 [9168, 10,169]
% Right	48.6	45.9	48.6	48.6	51.4
% Bilateral	48.6	54.1	48.6	48.6	43.2
% Left	2.7	0	2.7	2.7	5.4
*r* with POI average	NA	0.99 [0.97, 1]	0.99 [0.97, 0.99]	0.96 [0.92, 0.98]	0.96 [0.92, 0.98]
Teddy bear picnic					
Mean LI	−1.61 [−2.17, −1.06]	−1.62 [−2.17, −1.06]	−0.86 [−1.15, −0.57]	−1.04 [−1.39, −0.68]	−1.04 [−1.39, −0.68]
Within‐subject SE LI	0.80 [0.71, 0.89]	0.79 [0.70, 0.88]	0.61 [0.56, 0.66]	0.55 [0.50, 0.59]	0.29 [0.27, 0.31]
*R* ^2^	NA	NA	0.06 [0.05, 0.08]	0.20 [0.17, 0.23]	0.76 [0.74, 0.79]
AIC	NA	NA	8552 [8095, 9009]	8321 [7883, 8760]	6664 [6297, 7031]
BIC	NA	NA	8599 [8142, 9056]	8396 [7957, 8834]	7499 [7120, 7879]
% Right	29.7	27	21.6	35.1	43.2
% Bilateral	67.6	70.3	78.4	62.2	54.1
% Left	2.7	2.7	0	2.7	2.7
*r* with POI average	NA	0.99 [0.98, 1]	0.92 [0.82, 0.96]	0.94 [0.86, 0.97]	0.94 [0.86, 0.97]

Abbreviations: AIC, Akaike information criterion; BIC, Bayesian information criterion; GAM, generalised additive models; GLM, generalised linear models; LI, laterality index; POI, period of interest; SE, standard error.

**FIGURE 5:**
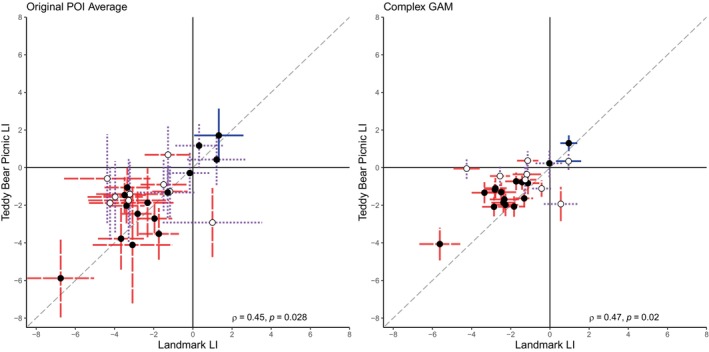
Scatterplots of laterality indices from the POI averaging and complex GAM methods for the visuospatial tasks from the adult dataset. GAM, generalised additive models; POI, period of interest. Scatterplots show the relationship between the landmark and teddy bear picnic LIs for the adult dataset when estimated using the original POI averaging method (left) and the complex GAM method (right). Spearman correlations and *p*‐values for both plots are included. Error bars show the 95% CI for each participant, coded by colour and pattern to indicate categorisation of each LI as either left‐lateralised (unbroken blue line), right‐lateralised (dashed red line), or bilateral (dotted purple line). Black circles indicate that the adult and child‐friendly task have the same categorisation, and white circles indicate different lateralisation categories for the two tasks.

Figure 5 presents scatterplots of the relationship between the landmark and teddy bear picnic LIs for the original POI averaging method and the complex GAM method. The Spearman correlation for the two tasks using the original method was significant, *ρ*(22) = .45, *p* = .028, as was the correlation using the complex GAM method, *ρ*(22) = .48, *p* = .020. After categorising participants for the visuospatial tasks, 62.5% of participants (*n* = 15) have the same categorisation when using the original POI averaging method, whereas 66.7% of participants (*n* = 16) have the same categorisation for both tasks using the complex GAM method. The relationship between the landmark and teddy bear picnic categorisations according to the complex GAM method failed to reach significance, *ρ*(22) = .38, *p* = .072.

### 3.2 Child dataset

**FIGURE 6:**
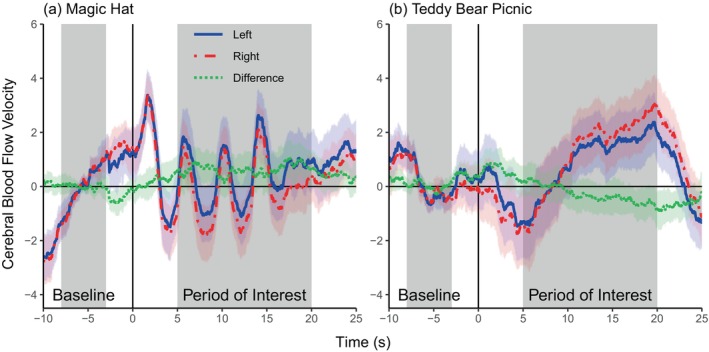
Group‐averaged change in blood flow velocity for the child dataset. Blood flow velocity change for the left (unbroken blue line) and right (dashed red line) middle cerebral arteries for the magic hat task (a) and teddy bear picnic task (b). The left‐minus‐right difference is shown as the green dotted line. Shaded ranges represent the 95% CI. The period of interest (POI) and baseline period (used for the POI averaging method) are shown in grey.

Group‐averaged blood flow velocity waveforms for the magic hat and teddy bear picnic tasks are shown in Figure 6. For the 50 participants with 8 or more acceptable epochs for the magic hat task, the median number of epochs was 15 (IQR = 6, minimum = 8, maximum = 18). For the 43 participants with 8 or more acceptable epochs for the teddy bear picnic task, the median number of epochs was 14 (IQR = 6, minimum = 8, maximum = 18).

**FIGURE 7:**
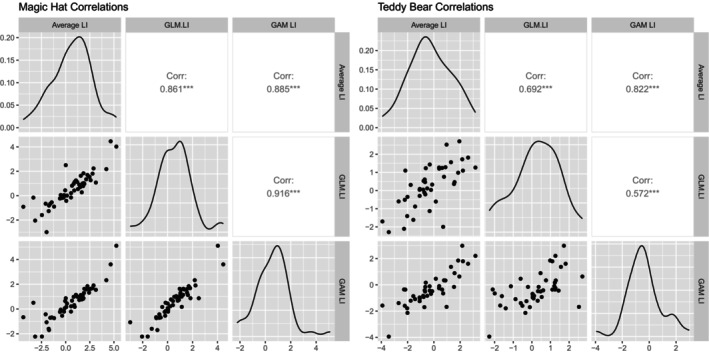
Correlations matrices, scatterplots and density plots comparing different processing methods for the child dataset. GAM, generalised additive model; GLM, generalised linear model; LI, laterality index.

Figure 7 presents the correlation matrices for the LIs produces by the different methods for both tasks, and Table 3 presents the model fit descriptors for the magic hat and teddy bear picnic task respectively. The complex GAM method is strongly correlated with the POI averaging method for both tasks (*r* > .8), produces a mean standard error of individual LIs less than half the standard error for the POI averaging method, and had the best model fit according to the *R* ^2^, AIC, and BIC values out of the GLM/GAM models for both tasks. The mean LIs show a general bias towards the expected hemisphere for both tasks at the group level for the complex GAM method. As with the adult datasets, the complex GAM method gives the smallest percentage of bilateral cases for both tasks. Spearman rank‐order correlations were used to determine if LIs for either task differed by age in the child dataset. LIs for the magic hat task did not correlate with age in years for the POI averaging method, *ρ*(48) = 0.24, *p* = .098, or the complex GAM method, *ρ*(48) = 0.22, *p* = .124; LIs for the teddy bear picnic task also did not correlate with age in years for the POI averaging method, *ρ*(41) = −0.09, *p* = .580, or the complex GAM methods, *ρ*(41) = 0.12, *p* = .459.

**TABLE 3:** Mean (95% CI) for indicators of model fit for the child dataset.

Index	POI average method	GLM method	Simple GAM method	Complex GAM method
Magic hat				
Mean LI	0.62 [0.08, 1.16]	0.56 [0.20, 0.92]	0.56 [0.21, 0.92]	0.56 [0.21, 0.92]
Within‐subject SE LI	1.10 [0.92, 1.28]	0.82 [0.74, 0.90]	0.73 [0.66, 0.80]	0.47 [0.41, 0.53]
*R* ^2^	NA	0.04 [0.03, 0.05]	0.12 [0.10, 0.14]	0.64 [0.60, 0.67]
AIC	NA	9033 [8426, 9639]	8921 [8317, 9525]	7692 [7183, 8201]
BIC	NA	9079 [8473, 9686]	9010 [8405, 9615]	8287 [7744, 8830]
% Right	5.6	5.6	7.4	9.3
% Bilateral	59.3	75.9	68.5	46.3
% Left	35.2	18.5	24.1	44.4
*r* with POI average	NA	0.86 [0.77, 0.92]	0.88 [0.8, 0.9]	0.88 [0.8, 0.93]
Teddy bear picnic				
Mean LI	−0.28 [−0.71, 0.16]	0.25 [−0.07, 0.57]	−0.35 [−0.69, −0.01]	−0.35 [−0.69, −0.01]
Within‐subject SE LI	1.07 [0.79, 1.36]	0.89 [0.82, 0.96]	0.76 [0.71, 0.82]	0.42 [0.37, 0.46]
*R* ^2^	NA	0.05 [0.04, 0.06]	0.14 [0.12, 0.17]	0.73 [0.70, 0.77]
AIC	NA	9081 [8470, 9692]	8949 [8337, 9562]	7330 [6820, 7839]
BIC	NA	9128 [8516, 9739]	9032 [8418, 9645]	7957 [7412, 8502]
% Right	20.4	3.7	20.4	27.8
% Bilateral	66.7	94.4	72.2	59.3
% Left	13	1.9	7.4	13
*r* with POI average	NA	0.69 [0.49, 0.82]	0.82 [0.69, 0.9]	0.82 [0.69, 0.9]

Abbreviations: AIC, Akaike information criterion; BIC, Bayesian information criterion; GAM, generalised additive models; GLM, generalised linear models; LI, laterality index; POI, period of interest; SE, standard error.

## 4 DISCUSSION

The aim of this paper was to assess the use of recent GLM and GAM methods reported by Thompson et al. (2023) for estimating LIs with adult fTCD data collected using child‐friendly language and visuospatial lateralisation tasks, and to evaluate their use with fTCD data from young children. Our findings show that the complex GAM method, which included epoch‐related variation as a predictor, substantially decreased the SE of the LIs compared to all other methods tested while showing a strong association with LIs generated by the POI averaging method for both the adult and child samples. The complex GAM method also showed better model fit measurements than the GLM and simple GAM methods for all tasks for both adult and child participants.

The correlations between the child‐friendly tasks and their corresponding adult task exceed the .40 threshold set in Quin‐Conroy et al. (2022) for determining validity. This supports the magic hat and teddy bear picnic tasks as valid measures of language and visuospatial lateralisation when the fTCD data is processed using GAM. There was notable decrease in the number of participants with the same categorisation for the language tasks for the complex GAM method compared to the original POI averaging method (from 72.2% to 55.6%), which is likely caused by the increase in participants categorised as left‐lateralised for the word generation task which was not mirrored to the same extent for the magic hat task. This could be a result of the additional non‐task‐related stimuli in the magic hat task compared to the word generation task, which is necessary to increase engagement for young children completing the task, although it should be noted that more participants were categorised the same for the two visuospatial tasks when using the complex GAM method. It is also possible that the word generation and magic hat tasks might lateralise differently in some individuals; Bradshaw et al.'s (2017) systematic review showed that verbal fluency tasks, such as the word generation task, produce a stronger left‐lateralised response than picture naming tasks. However, picture naming tasks are more feasible for young children or children with language delays, making them a more appropriate option for these populations despite their weaker left‐hemispheric response compared to verbal fluency. As the magic hat task shows the expected left‐lateralised bias for both samples in the current paper and the LIs for the magic hat task are strongly correlated with the word generation LIs for the adult sample reported here, the evidence supports the task as a suitable measure of language lateralisation for children, particularly when lateralisation is analysed using LIs (e.g., see the regression analyses by Lust et al., 2011) as opposed to a left/right/bilateral categorisation.

In sum, our analyses have demonstrated that the GAM model‐based method of processing fTCD data generates more precise LIs compared to the POI averaging method for data from young children. We recommend future research using fTCD with young children consider using GAM to reduce the noise in their LI estimates.

## CONFLICT OF INTEREST STATEMENT

The authors declare no conflicts of interest.

## Data Availability

The data that support the findings of this study are openly available in Open Science Framework at https://osf.io/2n5gj/.
